# Authors' reply

**Published:** 2009-10

**Authors:** S. Jasuja, A. K. Gupta, R. Choudhry, V. Kher, D. K. Aggarwal, A. Mishra, M. Agarwal, A. Sarin, M. K. Mishra, V. Raina

**Affiliations:** Department of Nephrology, Delhi, India; 1 Department of Nephrology, AHERF, Delhi, India; 2 Department of Nephrology, Fortis Hospitals, Delhi, India; 3 Department of Nephrology, Indraprastha Apollo Hospital, Delhi, India; 4 Department of Immunology and Molecular Biology, Delhi, India

Sir,

We appreciate the critical comments made by Dr. Agarwal.[1] The highlight of our article is that approximately 30% of patients in dialysis will not be diagnosed HCV+ve with the currently available 3^rd^ generation Elisa.[2]

We will try to clarify the points raised as they appear in the letter to the editor.

1. We agree that there is significant literature available on HCV from Indian centers. However, most of these do not utilize HCV RNA for diagnosis. We related our observations to studies using similar techniques based on PCR. Majority of the references quoted are out of context because of methodology,[5,6,8,9,10,11,12,14] non-relevant group studies[7,15,16,17] or review article.[13]
2. Duration of ESKD/ESRD, whether on HD or renal transplant, is a risk factor for HCV.[3,4,18]
3. On treatment, HCV patients are excluded because this may affect results of HCV PCR. In fact, some positive patients on treatment were HCV RNA negative at the time of our study. (These were not part of the study)
4. We do not separate HCV patients but follow universal precautions, while HCV dialyzers are reprocessed in a separate area. However, this has not been the focus of our article.
5. This study looks at point prevalence of HCV and was neither prospective nor retrospective. This was done in 2005 over a two-week period.
6. The numbers in the table I reflect not the doses but the number of courses taken by the patients. Twice or more vaccination implies repeating the entire vaccination schedule comprising of four doses, each time twice or more. We agree that hepatitis B vaccination does not in any way affect HCV prevalence. We purely mentioned it as another variable associated with population under study.
7. Refer to point 5.
8. Duration of CKD was estimated by the history recall and patient records.
9. Since our study was neither longitudinal nor cohort, it is not possible to calculate the probability of an individual that the reader suggested from this data. The prevalence of HCV positivity was 7.4% in patients who had duration of dialysis less than or equal to 16 months and prevalence of HCV positive is 45.2% for those who had more than 16 months of duration of dialysis. The change in prevalence from 7.4% to 45.2% was not in just one month. ROC curve is appropriate here, because duration of dialysis can be used as diagnostic tool for HCV. The sum of sensitivity and specificity at cut-off more than 16 months was higher; sensitivity (87.5%) and specificity (60%) seems appropriate.
10. The multivariable logistic regression was used to find the potential risk factors for HCV positivity and duration of dialysis was one of the potential risk factor. The results showed one month increase in duration of dialysis, increased odds 1.06 times to have HCV RNA positivity after adjusted for other potential risk factors.
11. Anti tubercular drugs and their effects were not looked into.
12. ALT has been used as a surrogate marker for HCV infection though its correlation has been poor. In our study, low serum albumin, high ALT were significant correlates for HCV RNA positivity. In fact, the normal cut offs for ALT in dialysis patients is much lower than that amongst non-dialysis patients. It only suggests that those with higher ALT had higher chances of HCV RNA positivity.
13. CDC does not recommend isolation of HCV+ve patients. We in our unit also do not isolate patients. We have not made any comments on whether it should be recommended or not. It is a matter of debate in the literature too. This has also not been the focus of our article.
